# Genetic and morphometric divergence in threespine stickleback in the Chignik catchment, Alaska

**DOI:** 10.1002/ece3.918

**Published:** 2013-12-18

**Authors:** Annette Taugbøl, Claudia Junge, Thomas P Quinn, Anders Herland, Leif Asbjørn Vøllestad

**Affiliations:** 1 Centre for Ecological and Evolutionary Synthesis (CEES)Department of Biosciences, University of OsloP.O.Box 1066, Blindern, NO-0316, Norway; 2 School of Aquatic and Fishery Sciences, University of WashingtonBox 355020, Seattle, Washington, 98195-5020

**Keywords:** Adaptation, hybridization, life-history polymorphism, microsatellites, phenotypic differentiation, population differentiation

## Abstract

Divergent selection pressures induced by different environmental conditions typically lead to variation in life history, behavior, and morphology. When populations are locally adapted to their current environment, selection may limit movement into novel sites, leading to neutral and adaptive genetic divergence in allopatric populations. Subsequently, divergence can be reinforced by development of pre-or postzygotic barriers to gene flow. The threespine stickleback, *Gasterosteus aculeatus*, is a primarily marine fish that has invaded freshwater repeatedly in postglacial times. After invasion, the established freshwater populations typically show rapid diversification of several traits as they become reproductively isolated from their ancestral marine population. In this study, we examine the genetic and morphometric differentiation between sticklebacks living in an open system comprising a brackish water lagoon, two freshwater lakes, and connecting rivers. By applying a set of microsatellite markers, we disentangled the genetic relationship of the individuals across the diverse environments and identified two genetic populations: one associated with brackish and the other with the freshwater environments. The “brackish” sticklebacks were larger and had a different body shape than those in freshwater. However, we found evidence for upstream migration from the brackish lagoon into the freshwater environments, as fish that were genetically and morphometrically similar to the lagoon fish were found in all freshwater sampling sites. Regardless, few F1-hybrids were identified, and it therefore appears that some pre-and/or postzygotic barriers to gene flow rather than geographic distance are causing the divergence in this system.

## Introduction

The genetic structure of contemporary populations is the result of both historical and current ecological and evolutionary processes. Habitats are often not stable over evolutionary timescales, and as environments change, organisms adapt, perish, or disperse. During the last ice age, much of the freshwater habitats in North America and Eurasia were inaccessible due to an extensive sheet of ice (the last glacial maximum was ∼18,000 years ago (Clark et al. 2009)). As the ice retreated, new freshwater habitats became accessible and were colonized by fish and other freshwater organisms expanding from glacial refuges, either through migration corridors (rivers and lakes) or through coastal dispersal (Lindsey and McPhail 1986, 1986). Such coastal dispersal is also a contemporary process in some areas (Milner and York 2001; Milner et al. 2008). Dispersal through marine waters is especially prevalent for anadromous and euryhaline fishes, such as salmonids (*Oncorhynchus*,*Salmo*, and *Salvelinus* spp.) (Hendry et al. 2004) and the threespine stickleback, *Gasterosteus aculeatus*.

The threespine stickleback (hereafter stickleback) has invaded many young, postglacial habitats through coastal dispersal (Bell and Foster 1994; Klepaker 1995; Von Hippel and Weigner 2004) and is today found in a wide variety of marine, brackish, and freshwater environments (Wootton 1976; Bell and Foster 1994). Following freshwater invasion, they have diverged in many phenotypic traits compared to the ancestral marine ecotype (Bell 1977; Klepaker 1993; McKinnon and Rundle 2002), making it a model species in evolutionary biology. One phenotypic trait that commonly differs between the marine and freshwater stickleback is body size; marine sticklebacks tend to be larger than those in freshwater (McPhail 1994), possibly resulting from a combination of environmental and genetic factors (Jones et al. 2012). Body size seems to be an important trait for mate choice for the stickleback (McKinnon et al. 2004; Conte and Schluter 2012), potentially functioning as a prezygotic barrier to gene flow between marine and freshwater fish. Another well-described divergent trait in stickleback is the number and location of lateral plates, which vary within and among populations (Hagen 1967; Narver 1969; Hagen and Gilbertson 1972; Hagen and Moodie 1982; Klepaker 1996). On the basis of the location of the plates, a stickleback can be assigned into one of three commonly recognized forms: complete-, partial-, and low-plated morphs (Wootton 1976). The different lateral plate morphs are typically found in different salinity environments, with the complete-, the partial-, and the low-plated morphs being associated with high, intermediate, and low salinity, respectively (Heuts 1947; Münzing 1963; Wootton 1976). Recent findings indicate that the repeated loss of lateral plates across different freshwater populations occurred as a consequence of parallel directional selection on one major locus, the *Ectodysplasin* (*Eda*) gene (Colosimo et al. 2005), as allele variants of this gene are strongly linked to the lateral plate morphs (Colosimo et al. 2005; Le Rouzic et al. 2011; Jones et al. 2012).

**Figure 1:**
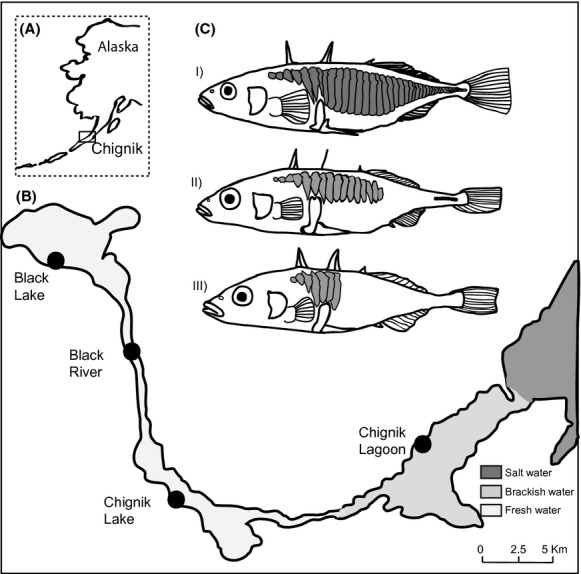
Study area. (A) Map of Alaska showing the position of the Chignik Lake system. (B) Locations of the four sampling sites, the graded color is according to salinity. (C) Figure of the threespine stickleback morph variation found within the Chignik system, I) completely, II) partial, and III) low plated.

In this study, we investigated gene flow between stickleback populations inhabiting a southwestern Alaskan lagoon-river system (Fig. 1). Movement between environments differing in salinity is physiologically costly, and salinity gradients can therefore limit gene flow in fishes (Moyle and Cech 1996). Previous studies from this system showed that stickleback from the brackish lagoon were monomorphic for the completely plated morph, whereas the freshwater sites had all of the three lateral plate morphs. Further, the completely plated stickleback in the marine lagoon differed from the freshwater fish by having more lateral plates and a more developed keel (Narver 1969). The fish in the opposing environments were also reported to have different life histories; the lagoon population matured at one year of age and bred in *Zostera* (eel grass) belts in the brackish water and in the lower parts of the freshwater habitat, whereas the freshwater fish bred in freshwater habitats at age two (Narver 1969). Apparently, all these fish die after breeding as no older age classes were recorded for either population (Narver 1969). Intensive upstream spring migrations between lakes have been observed (Narver 1969; Harvey et al. 1997), indicating that there are no physical barriers to migration and hence potential for gene flow between the different environments. In this system, the differences in morphology and life history could have evolved due to reduced gene flow, in combination with different adaptations to the ecological environments as natural selection can generate phenotypic and genetic differences between populations (Schluter 2000, 2009; Nosil 2012). Alternatively, there could be one large, diverse population with some individuals that migrate between habitats, and variation in growth potential (likely higher in marine than freshwater environments) and selective predation on low plate morphs in marine waters causing the observed differences in body size and shape.

The main goals of this study were to (1) examine the genetic population structure and integrity within the study system (i.e., determine how many distinct populations are present and identify potential hybrids) and (2) determine how size and shape differ between fish from the brackish and freshwater habitats. We screened 14 neutral microsatellite markers and tested for genetic relationships in fish sampled at four different locations – a brackish water lagoon, a river, and two lakes in the Chignik system in Alaska (Fig. 1). Subsequently, we tested for phenotypic differences in body shape among the detected groups based on 30 digitized landmarks. Our analyses revealed both migrants and hybrids between two well-defined genetic populations, and it was therefore particularly interesting to test for morphological differences between these individuals and the resident, nonhybrid individuals from the freshwater and lagoon habitats.

## Materials and Methods

### Study area and stickleback collection

Adult threespine sticklebacks (length >4 cm, when all the lateral plates are fully developed (Bell 1981)) were collected from the Chignik Lake system of southwestern Alaska (56°25′ 40″N, 158°75′ 60″W) (Fig. 1A,B), using beach seines (35 × 4 m, 3 mm mesh), tow nets (1.8 × 2.7 m), and fyke nets (1.22 m^2^ frame with 3–5 m wings). At each of four locations, the fish were collected from a single site, with sample sizes between 78 and 122 individuals. All sampling was conducted in the two last weeks of June 2009, within the breeding season for stickleback. The sampled fish were stored in 95% ethanol.

The sample locations were all areas where sticklebacks are very abundant: Chignik Lagoon, Chignik Lake, the Black River, and Black Lake (Fig. 1B). The Chignik Lagoon is a semi-enclosed estuary ranging about 12 km from Chignik Bay up to the Chignik River. Depending on the location in the lagoon and the stage of the tide, the salinity ranges from 0 to about 30‰ (Simmons et al. 2013). Tidal amplitudes that exceed 3 m can expose half the estuarine substrate, largely covered by eelgrass (*Zostera spp*.). The sample was collected from a site in the middle of the lagoon, between the outlet of the Chignik River and the sand spit that separates the lagoon from the more oceanic Chignik Bay. The Chignik River (7.2 km long) drains Chignik Lake (22 km^2^), a deep lake (maximum depth of 64 m) with a shoreline dominated by gravel. The Black River (12 km) connects Chignik Lake to Black Lake, which is larger (41 km^2^) but shallower (maximum depth 4 m) than Chignik Lake. Black Lake rapidly warms up in the spring and is highly productive with abundant vegetation and provides good breeding habitat for threespine stickleback (Narver 1969). The fish communities of these two lakes are dominated numerically by threespine sticklebacks and juvenile sockeye salmon, *Oncorhynchus nerka* (Westley et al. 2010). The main fish predators are juvenile coho salmon (*O. kisutch*) and Dolly Varden (*Salvelinus malma)* (Roos 1959; Narver and Dahlberg 1965; Ruggerone 1992).

### DNA extraction, PCR amplification, and genotyping

Genomic DNA was extracted from a pectoral fin from each fish using the salt-extraction method developed by Aljanabi and Martinez (1997). A total of 14 potentially neutral and two quantitative trait loci (QTL) microsatellite markers (Appendix S1) were genotyped for 389 individuals; 104 from the Chignik Lagoon, 122 from Chignik Lake, 85 from Black River, and 78 from Black Lake. This set of markers was selected to identify potential genetic structure within or across the populations and to discriminate plate morphs (stn382) and sex (idh). Each PCR had a total volume of 6 *μ*L, where each mixture contained 1–5 ng of genomic DNA, 1 × *Q* multiplex PCR solution (Qiagen, Hilden, Germany), and 1 pmol of each primer. The forward primers were fluorescently labeled based on their fragment lengths and the complete multiplex (Appendix S1). The PCR profiles for the 14 neutral markers were divided up into three multiplexes and consisted of 95°C for 15 min, followed by 37 cycles of 94°C for 30 sec, 59°C for 90 sec, 72°C for 60 sec, an extension step at 60°C for 30 min and a final extension step at 20°C for 10 min. The PCR products were diluted, and 1 *μ*L of that dilution was added to a mixture of 10 *μ*L formamide and 0.125 *μ*L allelic size standard (LIZ 500 bp, Applied Biosystems, ABI, Foster City, CA) for electrophoresis on a 3730 DNA Analyzer (ABI). The software GENEMAPPER (ABI) was used to analyze the individual alleles through visual inspections and manual corrections. Neutrality was checked for all the 14 microsatellites in LOSITAN (Beaumont and Nichols 1996; Antao et al. 2008), testing both the stepwise mutation model and the infinite allele model using 5000 simulations at a false discovery rate of 0.1. For two of the microsatellites, stn309 and stn319, a weak signal of positive selection was detected for both models (*F*_ST_ 0.053 and 0.043 for stn309 and stn319, respectively), but including or excluding these markers did not qualitatively change the results of the population genetic structure (data not shown), and they were kept in the dataset as neutral markers for all the analyses.

The two quantitative trait loci *stn382* and *Idh* were run in simplexes. The marker *Stn*382 is located within intron one of the *Ectodysplacin* (*Eda*) gene on linkage group IV (Colosimo et al. 2005). This marker has two alleles that are highly correlated with the three recognized stickleback morphs (Colosimo et al. 2005). The homozygous “AA” is mostly associated with the completely plated, the “Aa” mostly with the partial plated, and the “aa” mostly with the low-plated morph. The amplification reactions for this locus were performed as described in Colosimo et al. (2005). As this marker has two alleles only, with fragment lengths of either 151 (“a”) or 218 (“A”) base pairs (bp), the individual genotype could be visualized on a 2% agarose gel. Fragment size was verified with a size standard (Generuler, Fermentas) and internal gel controls for the three genotypes. Sex determination of the fish was carried out genetically, using the *Idh* locus (Peichel et al. 2004). Two alleles are recognized, where females are homozygous for one of the alleles (allele size 302 bp), while males are heterozygous (allele sizes 271 bp and 302 bp). The alleles were also separated on a 2% agarose gel with internal positive controls.

### Population genetic structure analysis

Using sampling sites as proxies for “populations” might give a false impression of the actual population structure, especially if dispersal between sites is common or if multiple populations occupy a site. As sticklebacks have been observed migrating between lakes and rivers in the Chignik system (Harvey et al. 1997), we used a genetic self-assignment test to allocate all sampled individuals back to an unknown number of genetic clusters (“populations”) using the program STRUCTURE 2.3 (Pritchard et al. 2000, 2007). By running STRUCTURE without a priori sampling information, the program clusters individuals based on their allele frequencies alone by identifying putative groups in the data that minimize departure from Hardy–Weinberg equilibrium (HWE). We first ran an initial analysis in STRUCTURE, with correlated allele frequencies and LOCPRIOR (Hubisz et al. 2009), to test for the number of separate genetic units (*K *=* *1 to *K *=* *6; set manually) in our total sample. The admixture model probabilistically assigns each individual to one or more clusters (*K*) and estimates the proportion of ancestry (*Q*) to each cluster (ranging from zero to one). Values of *Q* can subsequently be used to assign individuals to genetic clusters irrespective of their sampling locations. We ran five independent analyses for each value of *K*, using 700,000 iterations (following a burn-in period of 500,000) (Pritchard et al. 2000). The number of *K* that best fits the data is estimated by comparing the log likelihood of the data given the number of clusters (lnP(X∣*K*)) (Pritchard et al. 2007). As using lnP(X∣*K*) criteria can lead to an overestimation of population numbers (Pritchard et al. 2007), we also examined the second-order rate of change of lnP(X∣*K*) (Δ*K*), which is a more conservative approach (Evanno et al. 2005). Output files obtained from STRUCTURE were graphically summarized using R (R Development Core Team 2011). After running STRUCTURE on all the data (*n *=* *389), it was evident that *K *=* *2 gave the best fit, clearly separating the lagoon fish from most of the fish sampled from the freshwater sites. We also analyzed subsets of the data to further verify that *K *=* *2 was the model that best fitted the data (for the three freshwater sites individually in addition to all fish from freshwater pooled).

To detect migratory individuals, the fish were separated into lagoon or freshwater fish, based on whether their sampling site was brackish or fresh, and analyzed for putative migrants and individuals with recent immigrant ancestry using the assignment test implemented in STRUCTURE 2.3 (Pritchard et al. 2000). This test is a fully Bayesian method that uses sampling location as a prior when assigning the fish as migrants or admixed (hybrid) individuals. The program assumes a user-specified probability (*ν*) that corresponds to the likelihood of an individual being a migrant. To be conservative, we applied *ν *= 0.05 to our study, which corresponds to each individual having a 5% chance of being a migrant or having mixed ancestry. The model was run under the assumption of correlated allele frequencies among populations using a burn-in of 500,000 followed by 700,000 iterations. For all subsequent analyses, we assigned individuals as lagoon, migrants, hybrids, or freshwater fish, on the basis of their *Q*-value and migratory assignment from the STRUCTURE cluster at *K*_max_=2 (termed genetic population), in addition to using sampling sites directly for comparison.

To assess the population patterns and to characterize how differentiated the stickleback are in this region, we investigated the genetic diversity within and between the four sampling sites and the two genetically defined populations described earlier, including the individuals with recent migratory life history and putative hybrids. Genetic diversity (number of alleles per locus and sample), linkage disequilibrium (LD) of the markers, Hardy–Weinberg equilibrium (HWE), and observed and expected heterozygosity were calculated using Arlequin (Excoffier and Lischer 2010). Tests for significant deviations from HWE were performed for each locus and population. The p-values were estimated without bias using a Markov Chain (MC) random walk, following the algorithm of Guo and Thompson (1992), implemented in Arlequin (Excoffier et al. 2005). The MC parameters were set to default values, and corrections for multiple tests were performed by applying sequential Bonferroni corrections (Rice 1989). To compare the genetic differentiation between sampling populations and sampling populations excluding the migrant individuals, we calculated pairwise *F*_ST_ values for all pairs of populations using 10,000 permutations, and a significant level of *α *= 0.05 in the population comparison test implemented in Arlequin 3.5 (Excoffier and Lischer 2010).

### Morphological analyses

Fork length was measured to the nearest mm, and the lateral plates were counted directly on both sides of the body of each fish. The fish was classified as a complete-, partial-or low-plated morph according to Münzing (1963). To better recognize and place homologous landmarks (see below), each fish was stained in alizarin red (modified protocol after Dingerkus and Uhler (1977)), and a digital photograph was taken on the left side of each individual. The photograph was taken at a standardized distance, and a ruler was placed in each photograph for scaling. Females with bulky abdomens were excluded from the shape analysis. Further, the staining method also makes the fish very stiff, and some individuals were fixed in unnatural positions, making it hard to analyze their shape. After removing such individuals, 267 fish were analyzed for geometric shape variation.

**Figure 2:**
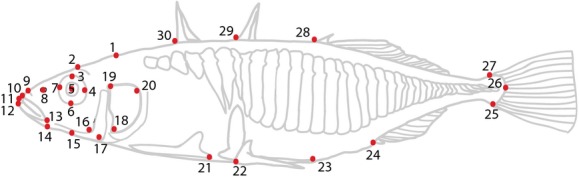
Outline of a threespine stickleback showing the locations of 30 landmarks (numbered circles) used to measure shape differences. The landmarks refer to: (1) Section between the frontal and the supraoccipital bone, identified as a small lowering in the head; (2) eye-brow; (3–7) eye; (8) nostril; (9) Maxilla; (10–12) anterior upper, middle, and lower lip; (13) posterior end of mouth; (14–15) outline of ventral jaw; (16) posterior jaw; (17) posterior preoperculum; (18–20) operculum; (21) posterior end of ectocoracoid; (22–23) anterior and posterior end of posterior process; (24) origin of anal spine; (25) insertion of ventral caudal fin ray; (26) posterior tail/end of vertebrae; (27) insertion of dorsal caudal fin ray; (28) origin of third spine; (29) origin of second spine; (30) origin of first spine.

To quantify geometric body shape variation in the genetically assigned stickleback populations, we placed 30 digitized landmarks on each picture (Fig. 2) using tpsDIG2 (Rholf 2005). The digitalized landmark positions were analyzed with MorphoJ (Klingenberg 2011), and figures were plotted in R (R Development Core Team 2011). We visualized the differences between the predefined groups by the use of a canonical variates analysis (CVA). CVA is a method that first performs a principal component analysis (PCA) of the pooled within-group variation to construct a coordinate system in which the position of each group can be positioned. After rescaling the axis proportionate to the elongation of the average fish, the program solves for the direction in which the fish seems to be farthest apart in the rescaled space by performing a PCA on the group centroids, producing the canonical variates (CVs). The scores of individuals on the CVs are the projection of the individuals onto these new coordinate axes (Zelditch et al. 2004). As all deviations from the centered data are expressed in the same metrics, it is possible to quantitatively visualize the shape change associated with a given principal component using warped outline drawings. As males and females may differ morphologically (Kitano et al. 2007), we tested for variation in shape within and between sex and genetic populations by extracting and plotting the two first axes of the CVA.

## Results

### Fish length and plate numbers at sampling locations

**Figure 3:**
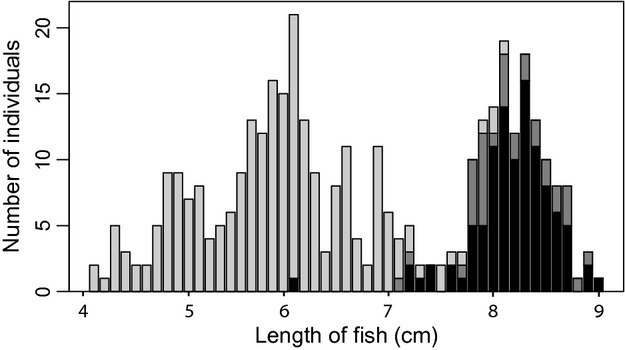
The length distribution for the fish sampled in freshwater (gray) and the lagoon (black). Light gray indicates a freshwater fish, dark gray indicates migrants; fish sampled in freshwater but with a genetic signature as a lagoon fish.

**Figure 4:**
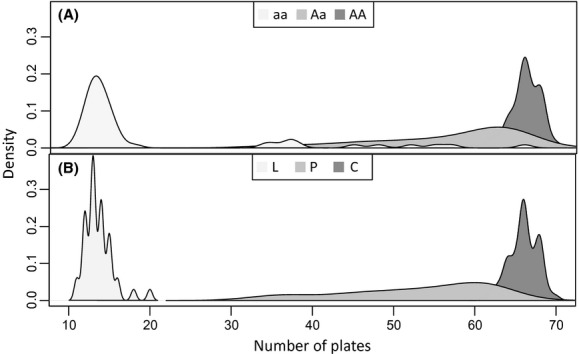
The frequencies of (A) the three *eda* genotypes: aa, Aa, and AA, and (B) the three morphs: complete (C), partial (P) and low (L) plated, in relation to total number of plates. As the separation of morph closely follows the distribution of the *eda* genotypes, only morph was used as an explanatory variable in the statistical analysis.

There were large differences in length and plate numbers among the four sampling sites (Table 1, Fig. 3). The fish from the lagoon were significantly larger (>1 cm on average) than fish from the freshwater sites (*F*_3, 385 _= 184, P < 0.001). Further, 82% of all sampled individuals were females (92%, 66%, 80%, and 93% females in Chignik Lagoon, Chignik Lake, Black River, and Black Lake, respectively). The females were on average 1 cm longer than the males (*F*_1, 368 _= 45.93, *P* < 0.001), and this pattern was seen at all sites. The fish sampled in the lagoon were all completely plated, whereas all three morphs were found in freshwater. Among the completely plated individuals, the lagoon fish had more plates than those collected in fresh water (an average of 61.5 in Chignik Lagoon and 60.9 in fresh water, after adjusting for length; *F*_2, 255 _= 76.72, P < 0.001). The freshwater samples contained high proportions of completely plated fish, even at the upper-most site, (Black Lake: 61%; Chignik Lake: 58%; Black River: 46%), and there were very few low-plated fish. There was no difference in plate number between the two sexes. Further, there was a tight linkage between the three *Eda* genotypes and the lateral plated morphs (Fig. 4); 78% of the low-plated individuals were aa, 66% of the partially plated were Aa, and 95% of the completely plated individuals were AA. Analyzing the data for either variable gave similar results, and therefore, only morph information was used as an explanatory variable in the subsequent statistical analyses.

**Table 1:** Mean length (cm) and plate number distributions for both males (M) and females (F) for completely (C), partially (P) and low plated (P) phenotypes at the four different sampling locations, in addition to their assigned genetic populations.

	Sample sites											
	Chignik Lagoon (*n *=* *104)			Chignik Lake (*n *=* *122)			Black River (*n *=* *85)			Black Lake (*n *=* *78)		
	C	P	L	C	P	L	C	P	L	C	P	L
Length												
M	7.2	–	–	5.5	4.8	5.0	6.6	5.9	6.0	6.5	–	6.0
F	8.2	–	–	5.9	5.3	5.5	7.1	6.1	5.8	7.0	6.2	6.7
#Plates												
M	67.8	–	–	65	55	14.2	67	50.5	12.7	65.2	–	14
F	66.9	–	–	65	55	14.4	65.3	51.9	13.2	65.6	51.6	12.8
	Genetically assigned individuals											
	Chignik Lagoon (*n *=* *104)			Migrants (*n *=* *35)			Hybrids (*n *=* *17)			Freshwater (*n *=* *234)		
C	P	L	C	P	L	C	P	L	C	P	L
Length												
M	7.2	–	–	7.36	–	–	–	–	–	5.5	5.5	5.4
F	8.2	–	–	8	–	–	6.8	6.2	–	6	5.9	5.9
#Plates												
M	67.8	–	–	67	–	–	–	–	–	64.9	52.3	13.6
F	66.8	–	–	67	–	–	65.8	58	–	64	52.59	13.4

### Descriptive statistics and population structure

The expected heterozygosity across the 14 neutral microsatellite loci varied between 0.582 and 0.944, with an average of 0.860, and the observed heterozygosity varied between 0.606 and 0.980, with an average of 0.827 (Appendix S2). There was no indication of linkage disequilibrium between any pairs of loci in any of the sample populations after Bonferroni corrections (*P *>* *0.05). Four loci deviated from HWE for some sampling sites, also after Bonferroni corrections; however, the pattern was not consistent across all population comparisons (Appendix S2). Therefore, all the 14 neutral microsatellite markers were used in all analyses.

**Figure 5:**
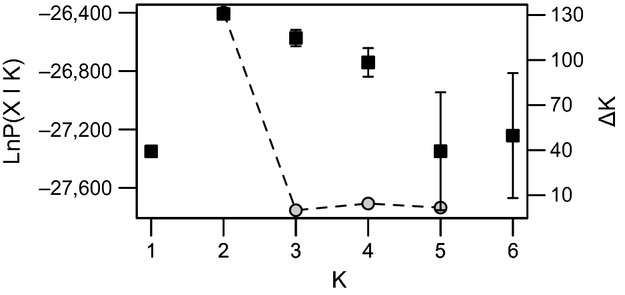
Interpretation of the number of genetic clusters (*K*) estimated in STRUCTURE. Both the likelihood of the data, lnP(*X*|*K*) (dark squares), and the standardized second-order rate of change of lnP(*X*|*K)*, the Δ*K* (gray circles), are plotted as a function of the assumed *K* (1–6) for each run. Each *K* has been run five replicated times, and the error bars for the lnP(*X*|*K*) indicate standard deviations.

**Figure 6:**
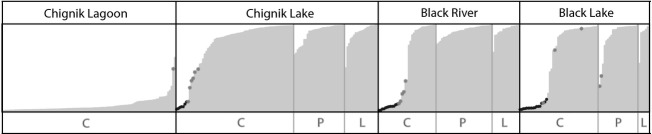
Summary plot of individual estimates of *Q*, where *Q* is a quantification of how likely each individual is belonging to each group (*K*) under consideration (here *K *=* *2). Each vertical line is one individual where the two colors represent individual membership to each cluster *Q*. black and grey dots indicate individuals identified as a first-generation migrant and as F1 hybrids, respectively. Sample sites are shown at the bottom, and the fish have been sorted based on location, morph (complete-[C], partial-[P] and low [L] plated) and *Q*-value.

The likelihood value lnP(X∣*K*) for each of the STRUCTURE runs without a priori sample information was highest for *K* = 2, indicating the presence of two genetic populations in this system. Visual inspection of the values indicated low variance for the replicated runs of *K *=* *1, 2, and 3 and increasing variance for *K *=* *4, 5, and 6 (Fig. 5). Additional evaluation of Δ*K* (Fig. 5)*,* and plotting individual *Q* values (Fig. 6) confirmed that *K *=* *2 captured the major genetic structure in the dataset.

### Migrants and genetic differentiation among populations

The STRUCTURE analysis for the detection of first-generation migrants and individuals with mixed ancestry identified 35 (32 females and 3 males) first-generation migrants from the lagoon in freshwater sites (7 in Chignik Lake, 12 in Black River, 16 in Black Lake), and 16 females with mixed ancestry (one in the lagoon, six in Chignik Lake, four in Black River, and five in Black Lake (Fig. 6)). This genetic identification of migrants and F1 hybrids was consistent with the morphological data. The length distributions of the migrants and lagoon fish did not differ, but the identified hybrids and the freshwater fish were on average 1.3 and 2.3 cm shorter than those in the lagoon, respectively (*F*_3, 385 _= 292.7, P < 0.001). All migrants were completely plated and had similar plate numbers as the lagoon fish, whereas completely plated hybrids and completely plated freshwater fish had an average of 0.8 and 1.7 fewer plates, respectively (ANCOVA with length as covariate; *F*_3, 253 _=_ _60.5, P < 0.001).

The STRUCTURE analysis revealed the presence of two genetic populations in the system, but *F*_ST_ tests indicated that the samples from the four sites were all significantly different from each other (Table 2; *F*_ST_ values from 0.003 to 0.046). The level of differentiation was highest between fish from the Chignik Lagoon and the Black River (*F*_ST _= 0.036), rather than between fish from the Chignik Lagoon and Black Lake (*F*_ST _= 0.028) as would have been expected in an isolation-by-distance scenario. When the individuals classified as migrants from the brackish environment were removed from the three freshwater samples, the level of differentiation between the lagoon sample and the respective freshwater samples increased (Table 2).

**Table 2:** Pairwise *F*_ST_ values between the sampling sites (above diagonal; dark gray) and between the sampling sites excluding the migrant individuals (below diagonal; light gray).

	Chignik Lagoon	Chignik Lake	Black River	Black Lake
Chignik Lagoon		0.034***	0.036***	0.028***
Chignik Lake	0.037***		0.005***	0.004***
Black River	0.046***	0.006***		0.004**
Black Lake	0.039***	0.003*	0.004**	

Significant pairwise comparison is indicated by

*** *P *<* *0.001;

** *P *<* *0.01;

* *P *<* *0.05.

### Geometric shape analysis

**Figure 7:**
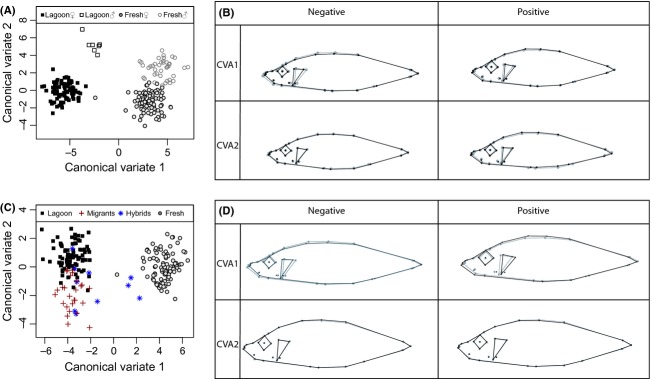
Geometric shape. (A) CVA scores of geometric shape for lagoon males (white squares), lagoon females (black squares), freshwater males (white circles), and freshwater females (gray circles); (B) The geometric shape changes for the two CVA axis, gray lines representing the average fish, black lines representing the landmark shifts associated with the vector values; (C) CVA scores for the genetically assigned populations, lagoon (black squares), migrants (red crosses), hybrids (blue stars), and freshwater (gray circles); D) the geometric shape changes for the two CVA axis.

Using 30 digitized landmarks on morphological traits (Fig. 2), we extracted geometric-morphometric information for the sticklebacks. As CVA analyses the relative positions of the groups in the sample, the method requires that the individuals be grouped before the analysis begins. We grouped the fish in two sets, one set including males and females from the lagoon and freshwater, excluding the identified migrants and hybrids (Fig. 7A), and another set including only female fish classified as either being lagoon fish, migrants, hybrids, or of freshwater origin (Fig. 7B). The comparison between the two sexes coming from the lagoon and freshwater clearly separated i) the two populations on the first axis (CV1) and ii) the two sexes on the second axis (CV2) (Fig. 7A). The lagoon fish had more streamlined bodies with thinner heads, smaller eyes, and more upward-pointing mouths (Fig. 7C) compared with the freshwater fish, and the females had more shallow bodies compared with the males. Visualizing the females separated into genetic populations also showed a clear separation of fish with a genetic signature from the lagoon and the freshwater environments (Fig. 7B) with the identified migrants grouping with the lagoon and the identified hybrids resembling both populations. The freshwater fish had larger eyes and a more bulky shape compared with the fish from the lagoon and the migrants (Fig. 7D). There was no evident separation between the three lateral plate morphs in geometric shape (results not shown).

## Discussion

The threespine stickleback in the Chignik system clustered into two distinct genetic populations: one associated with the lagoon environment and the other with the freshwater environments, indicating a significant barrier to gene flow at the freshwater–lagoon interface. Fish with a lagoon genetic signature were, however, commonly found in freshwater (5% of all samples in Chignik Lake, 14% in the Black River, and 20% in Black Lake, the uppermost site), but no fish with a distinct freshwater genetic signature was found in the Chignik Lagoon. We interpret these results as indicating that the main direction of gene flow in this system mirrors the evolutionary history of sticklebacks (i.e., from marine to freshwater habitats), rather than following the passive downstream direction. However, without more extensive sampling, especially at different locations in the lagoon and at different times of the year, this conclusion is tentative.

### Genetic variation and differentiation

Significant pairwise *F*_ST_ values were found between all four samples. However, the differentiation between the three freshwater samples was low, and when comparing all freshwater samples to the Chignik Lagoon sample, the *F*_ST_ values indicated very limited gene flow between these two environments. Moving between water with different salinities is costly for most fish, and salinity can therefore be a barrier to gene flow (Moyle and Cech 1996). However, the stickleback originated as a marine species (Bell 1977) and has repeatedly colonized freshwater habitats all over the northern hemisphere (Bell 1977), indicating that salinity itself does not prevent gene flow between adjacent stickleback populations differing in salinity levels (Grøtan et al. 2012). However, rapid parallel phenotypic radiations after colonization of freshwater habitats (Klepaker 1993; McKinnon and Rundle 2002) indicate that selection favors certain traits in the different environments. The differences between brackish water and freshwater stickleback observed in this study are consistent with other studies on sticklebacks. In a recent study from the Baltic Sea, absent of obvious physical barriers, the stickleback diverged in accordance with local differences in salinity (DeFaveri et al. 2013). Moreover, McCairns and Bernatchez (2008) studied threespine stickleback populations in the open St. Lawrence River system in Canada and found that the genetic differentiation (although weak) correlated more with salinity than with geographic distance. Thus, adaptation to different salinities may act as a barrier to gene flow after colonization occurs.

### Morphology

Freshwater colonization events have led to many changes in morphology between ancestral marine and derived freshwater sticklebacks (Bell 1977; McKinnon and Rundle 2002). The lagoon fish were significantly larger than the freshwater fish, consistent with findings from other studies showing that marine stickleback in general are larger than stickleback in freshwater (Wootton 1976; McKinnon et al. 2004). In the Chignik system, juvenile sockeye salmon utilizing similar habitats (the lagoon and the two freshwater lakes) also show increased growth rate and larger average overall body size in the lagoon (Simmons et al. 2013). These size differences may therefore be explained by increased growth potential in the marine environment relative to freshwater environments in the Chignik system (Bond 2013). Although there might be some biases associated with the local sampling, the size differences between freshwater and lagoon samples were marked and also consistent with work in the 1960s (Narver 1969).

Sticklebacks from the two environments differed in geometric shape. There are many examples of parallel morphological evolution in sticklebacks after colonizing freshwater habitats (McKinnon and Rundle 2002; Adachi et al. 2012) where the derived morphological variation is assumed to be adaptive (Bell 1977). Resource use during ontogeny influences morphology in stickleback populations (Day et al. 1994; Day and McPhail 1996; Kristjansson 2005) as well as in other fish species (Torres-Dowdall et al. 2012) and birds (Badyaev et al. 2002). Adaptation to benthic and limnetic food resources in many different species leads to specially adapted morphotypes (Schluter and McPhail 1992; Bernatchez 2004). Benthic and limnetic morphotypes are also common in the threespine stickleback (Larson 1976; McPhail 1992, 1994), and although the differentiation along this benthic–limnetic axis is generally continuous, a few stickleback populations have diverged into sympatric populations (species pairs) that feed exclusively on one prey type or the other (McPhail 1984). We have no direct evidence that the morphological divergence of the Chignik stickleback is driven by differential adaptation to food types. However, fish from the Chignik system seem to follow the benthic–limnetic divergence as the lagoon fish were more streamlined and had smaller heads than the more bulky freshwater fish (Schluter and McPhail 1992).

The observed phenotypic variation in this system could be resulting from genetic factors (McPhail 1977; Hendry et al. 2002; Leinonen et al. 2011; Jones et al. 2012) as the two populations are genetically differentiated, by phenotypic plasticity (Pfennig et al. 2010; McCairns and Bernatchez 2012), as they inhabit different habitats, or a combination of both factors. In a similar study spanning marine and fresh water environments McCairns and Bernatchez (2012) raised offspring in reciprocal salinities and found that most of the phenotypic divergence observed in the two original populations resulted from plastic responses to the environmental salinity rather than genetic differences in body shape. While we have no data on the underlying causes of morphological variation observed in these populations, it is likely that both genetic differentiation and plasticity are causing the observed geometric-morphometric shape differentiation in the two populations.

### Potential pre-and postzygotic barriers to gene flow

Divergent selection in different environments may lead to reproductive isolation through reduced gene flow and ultimately to ecological speciation (Schluter 2000, 2009; Nosil 2012). Hybridization and exchange of genes occur when allopatric species come in contact, or when reproductive isolation barriers break down between diverging species that still lack intrinsic genetic incompatibilities (Seehausen 2006). The two genetic stickleback populations in the Chignik system are differentiated morphologically, but there is potential for gene flow between populations as evidenced by individuals apparently of lagoon origin present in freshwater during the spawning period. However, the estimated level of hybridization was low; only 4.3% of the fish sampled in freshwater were genetically identified as F1 hybrids. This percentage is lower than reports from other hybrid zones; hybrid proportions of 46% and 33% were detected in the hybrid zones of Little Campbell River and River Thyne, respectively (Hagen 1967; Jones et al. 2006). However, in those studies, hybrids were identified based on lateral plate morphology alone (hybrids between completely plated marine and low-plated freshwater sticklebacks are usually partially plated), and this might underestimate the actual number of hybrids, as all the hybrids identified in the Chignik system were completely plated or overestimate the number of hybrids, as not all partially plated fish are hybrids (this study; Hagen and Moodie 1982).

The low hybridization rate observed in this study indicates the presence of pre-or postzygotic barriers to gene flow (De Cara et al. 2008). Adaptation to ecologically diverse environments can restrict gene flow between populations (Rundle and Nosil 2005), and natural selection against maladaptive hybrids reinforces premating isolation between sympatric species across taxa (Sætre et al. 1997; Rundle and Schluter 1998; Nosil et al. 2003; Singhal and Moritz 2012; Yukilevich 2012), including stickleback (Rundle and Schluter 1998). Phenotypically divergent populations inhabiting different ecological environments can experience selection against dispersers moving between them, limiting gene flow by mate preferences for similar phenotypes.

Body size (Nagel and Schluter 1998; McKinnon et al. 2004; Albert 2005; Conte and Schluter 2012) and shape (Head et al. 2013) appear to be an important trait for mate selection in sticklebacks and could be important also for the Chignik populations as they differ greatly in body size; both females and males from the lagoon were significantly larger than the freshwater fish. Positive assortative mating between conspecific members in areas where the migratory and resident freshwater forms coexist has been reported (Hay and McPhail 1975; McKinnon et al. 2004), and recent experiments indicated that body size alone functions as a mate signal between the morphologically different benthic and limnetic species pairs found in British Colombia (Conte and Schluter 2012), as could well be the case with the Chignik sticklebacks.

## Data Accessibility

Microsatellite, morphological and landmark data information: doi: 10.5061/dryad.t0b62.

## Supporting Information

**Appendix S1.** Table with descriptive statistics for the loci used to determine genetic background, *eda*-genotype and sex in this study. **Appendix S2.** Table with descriptive statistics for all individuals sampled at each sampling site and for the genetically assigned populations (see Materials and methods).

## References

[b1] Adachi T, Ishikawa A, Mori S, Makino W, Kume M, Kawata M (2012). Shifts in morphology and diet of non-native sticklebacks introduced into Japanese crater lakes. Ecol. Evol.

[b2] Albert AYK (2005). Mate choice, sexual imprinting, and speciation: a test of a one-allele isolating mechanism in sympatric sticklebacks. Evolution.

[b3] Aljanabi SM,, Martinez I (1997). Universal and rapid salt-extraction of high quality genomic DNA for PCR-based techniques. Nucleic Acids Res.

[b4] Antao T, Lopes A, Lopes RJ, Beja-Pereira A, Luikart G (2008). LOSITAN: a workbench to detect molecular adaptation based on a F(st)-outlier method. BMC Bioinformat.

[b5] Badyaev AV, Hill GE, Beck ML, Dervan AA, Duckworth RA, McGraw KJ (2002). Sex-biased hatching order and adaptive population divergence in a passerine bird. Science.

[b6] Beaumont MA,, Nichols RA (1996). Evaluating loci for use in the genetic analysis of population structure. Proceed. Royal Soc. B-Biol. Sci.

[b7] Bell MA (1977). Late Miocene marine threespine stickleback, *Gasterosteus aculeatus*, and its zoogeographic and evolutionary significance. Copeia.

[b8] Bell MA (1981). Lateral plate polymorphism and ontogeny of the complete plate morph of threespine sticklebacks *(Gasterosteus aculeatus)*. Evolution.

[b9] Bell MA,, Foster SA (1994). The evolutionary biology of the threespine stickleback.

[b10] Bernatchez L, Hendry AP, Stearns SC (2004). Ecological theory of adaptive radiation. An empirical assessment from Coregonine fishes (Salmoniformes). Evolution illuminated. Salmon and their relatives.

[b11] Bond MH (2013). Diversity in migration, habitat use, and growth of Dolly Varden char in Chignik Lakes, Alaska.

[b12] Clark PU, Dyke AS, Shakun JD, Carlson AE, Clark J, Wohlfarth B (2009). The last glacial maximum. Science.

[b13] Colosimo PF, Hosemann KE, Balabhadra S, Villarreal G, Dickson M, Grimwood J (2005). Widespread parallel evolution in sticklebacks by repeated fixation of ectodysplasin alleles. Science.

[b14] Conte GL,, Schluter D (2012). Experimental confirmation that body size determines mate preference via phenotype matching in a stickleback species pair. Evolution.

[b15] Day T, McPhail JD (1996). The effect of behavioural and morphological plasticity on foraging efficiency in the threespine stickleback (*Gasterosteus sp*. Oecologia.

[b16] Day T, Pritchard J, Schluter D (1994). A comparison of two sticklebacks. Evolution.

[b17] De Cara MAR, Barton NH, Kirkpatrick M (2008). A model for the evolution of assortative mating. Am. Nat.

[b18] DeFaveri J, Jonsson PR, Merila J (2013). Heterogeneous genomic differentiation in marine threespine stickleback: Adaptation along an environmental gradient. Evolution.

[b19] Dingerkus G,, Uhler LD (1977). Enzyme clearing of alcian stained whole small vertebrates for demonstration of cartilage. Stain Technol.

[b20] Evanno G, Regnaut S, Goudet J (2005). Detecting the number of clusters of individuals using the software STRUCTURE: a simulation study. Mol. Ecol.

[b21] Excoffier L,, Lischer HEL (2010). Arlequin suite ver 3.5: a new series of programs to perform population genetics analyses under Linux and Windows. Mol. Ecol. Res.

[b22] Excoffier L, Laval G, Schneider S (2005). Arlequin (version 3.0): an integrated software package for population genetics data analysis. Evol. Bioinformat.

[b23] Grøtan K, Østbye K, Taugbøl A, Vøllestad LA (2012). No short-term effect of salinity on oxygen consumption in threespine stickleback (*Gasterosteus aculeatus*) from fresh, brackish, and salt water. Can. J. Zool.

[b24] Guo SW,, Thompson EA (1992). Performing the exact test of Hardy-Weinberg proportion for multiple alleles. Biometrics.

[b25] Hagen DW, (1967). Isolating mechanism in threespine sticklebacks (*Gasterosteus*. J. Fish. Res. Board Can.

[b26] Hagen DW,, Gilbertson LG (1972). Geographic variation and environmental selection in *Gasterosteus-aculeatus* in Pacific Northwest, America. Evolution.

[b27] Hagen DW,, Moodie GEE (1982). Polymorphism for plate morphs in *Gasterosteus-aculeatus* on the east cost of Canada and an hypothesis for their global distribution. Can. J. Zool.

[b28] Harvey CJ, Ruggerone GT, Rogers DE (1997). Migrations of three-spined stickleback, nine-spined stickleback, and pond smelt in the Chignik catchment, Alaska. J. Fish Biol.

[b29] Hay DE,, McPhail JD (1975). Mate selection in threespine sticklebacks (*Gasteroseus*. Can. J. Zool.

[b30] Head ML, Kozak GM, Boughman JW (2013). Female mate preferences for male body size and shape promote sexual isolation in threespine sticklebacks. Ecol. Evol.

[b31] Hendry AP, Taylor EB, McPhail JD (2002). Adaptive divergence and the balance between selection and gene flow: Lake and stream stickleback in the misty system. Evolution.

[b32] Hendry AP, Castric V, Kinnison MT, Quinn TP, Hendry AP, Stearns SC (2004). The evolution of philopatry and dispersal: Homing versus straying in salmonids. Evolution illuminated. Salmon and their relatives.

[b33] Heuts MJ (1947). Experimental studies on adaptive evolution in *Gaserosteus-aculeatus L*. Evolution.

[b34] Hubisz MJ, Falush D, Stephens M, Pritchard JK (2009). Inferring weak population structure with the assistance of sample group information. Mol. Ecol. Res.

[b35] Jones FC, Brown C, Pemberton JM, Braithwaite VA (2006). Reproductive isolation in a threespine stickleback hybrid zone. J. Evol. Biol.

[b36] Jones FC, Grabherr MG, Chan YF, Russell P, Mauceli E, Johnson J (2012). The genomic basis of adaptive evolution in threespine sticklebacks. Nature.

[b37] Kitano J, Mori S, Peichel CL (2007). Sexual dimorphism in the external morphology of the threespine stickleback (*Gasterosteus aculeatus*. Copeia.

[b38] Klepaker T (1993). Morphological changes in a marine population of threespine sticklebacks, *Gasterosteus aculeatus*, recently isolated in freshwater. Canadian J. Zool.

[b39] Klepaker T (1995). Postglacial evolution in lateral plate morphs in Norwegian freshwater populations of threespine Stickleback (*Gasterosteus aculeatus*. Can. J. Zool.

[b40] Klepaker T (1996). Lateral plate polymorphism in marine and estuarine populations of the threespine stickleback (*Gasterosteus aculeatus*) along the coast of Norway. Copeia.

[b41] Klingenberg CP (2011). MorphoJ: an integrated software package for geometric morphometrics. Mol. Ecol. Res.

[b42] Kristjansson BK (2005). Rapid morphological changes in threespine stickleback, *Gasterosteus aculeatus*, in freshwater. Environ. Biol. Fishes.

[b43] Larson GL (1976). Social behavior and feeding ability of two phenotypes of *Gasterosteus aculeatus* in relation to their spatial and trophic segregation in a temperate lake. Can. J. Zool.

[b44] Le Rouzic A, Ostbye K, Klepaker TO, Hansen TF, Bernatchez L, Schluter D (2011). Strong and consistent natural selection associated with armour reduction in sticklebacks. Mol. Ecol.

[b45] Leinonen T, Cano JM, Merila J (2011). Genetics of body shape and armour variation in threespine sticklebacks. J. Evol. Biol.

[b46] Lindsey CC,, McPhail JD, Hocutt CH, Wiley EO (1986). Zoogeography of fishes of the Yukon and Mackenzie basins. The zoogeography of North American freshwater fishes.

[b47] McCairns RJS,, Bernatchez L (2008). Landscape genetic analyses reveal cryptic population structure and putative selection gradients in a large-scale estuarine environment. Mol. Ecol.

[b48] McCairns RJS,, Bernatchez L (2012). Plasticity and heritability of morphological variation within and between parapatric stickleback demes. J. Evol. Biol.

[b49] McKinnon JS,, Rundle HD (2002). Speciation in nature: the threespine stickleback model systems. Trends Ecol. Evol.

[b50] McKinnon JS, Mori S, Blackman BK, David L, Kingsley DM, Jamieson L (2004). Evidence for ecology's role in speciation. Nature.

[b51] McPhail JD (1977). Inherited interpopulation differences in size at first reproduction in threespine stickleback, *Gasterosteus aculeatus L*. Heredity.

[b52] McPhail JD (1984). Ecology and evolution of sympatric sticklebacks (*Gasterosteus aculeatus*)-morphological and genetic evidence for a species pair in Enos Lake, British-Colombia. Can. J. Zool.

[b53] McPhail JD (1992). Ecology and evolution of sympatric sticklebacks (*Gasterosteus*): evidence for a species-pair in Paxton Lake, Texada Island, British Columbia. Can. J. Zool.

[b54] McPhail JD, Bell AM, Foster JR (1994). Speciation and the evolution of reproductive isolation in the sticklebacks (*Gasterosteus*) of south-western British Columbia. The evolutionary biology of the threespine stickleback.

[b55] Milner AM,, York GS (2001). Factors influencing fish productivity in a newly formed watershed in Kenai Fjords National Park, Alaska. Archiv für Hydrobiologie.

[b56] Milner AM, Robertson KA, Veal AJ, Flory EA (2008). Colonization and development of an Alaskan stream community over 28 years. Front. Ecol. Environ.

[b57] Moyle PB, Cech JJ (1996). Fishes. An introduction to ichthyology.

[b58] Münzing J (1963). The evolution of variation and distributional patters in European populations of the three-spined stickleback, *Gasterosteus aculeatus*. Evolution.

[b59] Nagel L,, Schluter D (1998). Body size, natural selection, and speciation in sticklebacks. Evolution.

[b60] Narver DW (1969). Phenotypic variation in the threespine sticklebacks (*Gasterosteus aculeatus*) of the Chignik River system, Alaska. J. Fish. Res. Board Can.

[b61] Narver DW,, Dahlberg ML (1965). Estuarine food of Dolly Varden at Chignik, Alaska. Trans. Am. Fish. Soc.

[b62] Nosil P (2012). Ecological speciation.

[b63] Nosil P, Crespi BJ, Sandoval CP (2003). Reproductive isolation driven by the combined effects of ecological adaptation and reinforcement. Proceed. Royal Soc. B-Biol. Sci.

[b64] Peichel CL, Ross JA, Matson CK, Dickson M, Grimwood J, Schmutz J (2004). The master sex-determination locus in threespine sticklebacks is on a nascent Y chromosome. Curr. Biol.

[b65] Pfennig DW, Wund MA, Snell-Rood EC, Cruickshank T, Schlichting CD, Moczek AP (2010). Phenotypic plasticity's impacts on diversification and speciation. Trends Ecol. Evol.

[b66] Pritchard JK, Stephens M, Donnelly P (2000). Inference of population structure using multilocus genotype data. Genetics.

[b67] Pritchard J, Wen X, Falush D (2007).

[b501] R Development Core Team (2011). R: a language and environment for statistical computing.

[b68] Rholf FJ (2005). http://life.bio.sunysb.edu/morph/.

[b69] Rice WR (1989). Analyzing tables of statistical tests. Evolution.

[b70] Roos JF (1959). Feeding habits of the Dolly Varden, *Salvelinus malma* (Walbaum), at Chignik, Alaska. Trans. Am. Fish. Soc.

[b71] Ruggerone GT (1992). Threespine stickleback aggregation creates a potential predation refuge for sockeye salmon fry. Can. J. Zool.

[b72] Rundle HD, Nosil P (2005). Ecological speciation. Ecol. Lett.

[b73] Rundle HD,, Schluter D (1998). Reinforcement of stickleback mate preferences: sympatry breeds contempt. Evolution.

[b74] Sætre GP, Moum T, Bures S, Kral M, Adamjan M, Moreno J (1997). A sexually selected character displacement in flycatchers reinforces premating isolation. Nature.

[b75] Schluter D (2000). The ecology of adaptive radiation.

[b76] Schluter D (2009). Evidence for ecological speciation and its alternative. Science.

[b77] Schluter D,, McPhail JD (1992). Ecological character displacement and speciation in Sticklebacks. Am. Nat.

[b78] Seehausen O (2006). Conservation: losing biodiversity by reverse speciation. Curr. Biol.

[b79] Simmons RK, Quinn TP, Seeb LW, Schindler DE, Hilborn R (2013). Role of estuarine rearing for sockeye salmon in Alaska (USA). Marine Ecol.-Prog. Ser.

[b80] Singhal S,, Moritz C (2012). Strong selection against hybrids maintains a narrow contact zone between morphologically cryptic lineages in a rainforest lizard. Evolution.

[b81] Torres-Dowdall J, Handelsman CA, Reznick DN, Ghalambor CK (2012). Local adaptation and the evolution of phenotypic plasticity in Trinidadian guppies (*Poecilia reticulata*. Evolution.

[b82] Von Hippel FA,, Weigner H (2004). Sympatric anadromous-resident pairs of threespine stickleback species in young lakes and streams at Bering Glacier, Alaska. Behaviour.

[b83] Westley PAH, Schindler DE, Quinn TP, Ruggerone GT, Hilborn R (2010). Natural habitat change, commercial fishing, climate, and dispersal interact to restructure an Alaskan fish metacommunity. Oecologia.

[b84] Wootton RJ (1976). The biology of the sticklebacks.

[b85] Yukilevich R (2012). Asymetrical patterns of speciation uniquely support reinforcement in drosophila. Evolution.

[b86] Zelditch M, Swiderski D, Sheets H, Fink W (2004). Geometric Morphometrics for Biologists: A Primer.

