# Supplementary material for: Genetic and morphometric divergence in threespine stickleback in the Chignik catchment, Alaska
Source: Ecol Evol. 2013 Dec 18;4(2):144–56. doi: 10.1002/ece3.918 (PMC3925378; doi:10.1002/ece3.918)
Supplement: Supplementary file 1 — Appendix S1. Table with descriptive statistics for the loci used to determine genetic background, eda-genotype and sex in this study. Appendix S2. Table with descriptive statistics for all individuals sampled at each sampling site and for the genetically assigned populations (see Materials and methods). [file ece30004-0144-sd1.docx]

**Supporting information**

**Supporting information 1:** Table descriptive statistics for the loci used to determine genetic background, *eda*-genotype and sex in this study. Linkage group where the loci is located on the threespine stickleback genome; Primer, forward (F) and reverse (R) primer-sequences for the markers and the fluorescent labels used for multiplexing; Range of the alleles (in base-pairs), A indicate the number of alleles identified for each marker. H_O_, observed heterozygozity; H_E_, expected heterozygosity, F_ST_, fixation index; F_IS_, inbreeding coefficient.

| **Locus** | **LG** | **Primer** | **Range (A)** | **H_O_** | **H_E_** | **F_ST_** | **F_IS_** |
| --- | --- | --- | --- | --- | --- | --- | --- |
|  |  |  |  |  |  |  |  |
| Stn14^1^ | I | F: **NED-**GTGAACCAAACTCATAACAGCG | 185-216 (30) | 0.864 | 0.905 | 0.015 | 0.045 |
|  |  | R: CTTGTCCCTGGATGAGAACC |  |  |  |  |  |
| Stn61^1^ | VI | F: **FAM-**AGGAGGTCACCACAGGAGG | 94-144 (29) | 0.824 | 0.882 | 0.02 | 0.065 |
|  |  | R: GACGAGTCAGCAGTTTGAGC |  |  |  |  |  |
| Stn67^1^ | VI | F: **PET-**CTGGTTTAGTGGTGATGGGC | 160-260 (37) | 0.875 | 0.91 | 0.011 | 0.039 |
|  |  | R: GACGAGTCAGCAGTTTGAGC |  |  |  |  |  |
| Stn152^1^ | XIII | F: **FAM-**ATGGAATATCGACAGAGCCG | 186-270 (34) | 0.957 | 0.93 | 0.012 | -0.028 |
|  |  | R: ACGCCATAATCTCCATACCC |  |  |  |  |  |
| Stn175^1^ | XVI | F: **HEX-**TCTACATCATCTGTTACCACGG | 199-271 (28) | 0.851 | 0.839 | 0.02 | -0.014 |
|  |  | R: TTGCATGAGCGTGTAAAACC |  |  |  |  |  |
| Stn185^1^ | XIX | F: **NED-**GGAGGGTGAAGAACAACTGG | 146-188 (23) | 0.841 | 0.878 | 0.009 | 0.042 |
|  |  | R: GGAGAGCAGCAGAGATACGG |  |  |  |  |  |
| Stn186^1^ | XIX | F: **HEX-**CTCACTTCCCAAATGTCACG | 132-195 (32) | 0.761 | 0.827 | 0.025 | 0.079 |
|  |  | R: TACCTGGCAGCCTAATGACC |  |  |  |  |  |
| Stn211* | X | F: **FAM-**ACAACTCTTCCTTTGGCTGG | 101-139 (16) | 0.711 | 0.799 | 0.026 | 0.11 |
|  |  | R: ATAAATCCAGGCCACACACG |  |  |  |  |  |
| Stn232* | XVI | F: **NED-**ATGTGACCTGTAGGAAGCCG | 186-234 (35) | 0.862 | 0.869 | 0.008 | 0.008 |
|  |  | R: TCTGATGGGTGAGAAGACGC |  |  |  |  |  |
| Stn265^1^ | II | F: **FAM-**CGTAGTGTGAAACCACAGGC | 80-162 (40) | 0.913 | 0.932 | 0.013 | 0.02 |
|  |  | R: TGTGTCCACAGAGATGAGGC |  |  |  |  |  |
| Stn304* | XIII | F: **NED-**TCTAGCTCTTCTTCCAGGGC | 140-158 (10) | 0.65 | 0.695 | 0.053 | 0.065 |
|  |  | R: AGATGGCCCAGTTATGAACG |  |  |  |  |  |
| Stn319* | XII | F: **FAM-**CCCTCACTGATAACTAGGCCC | 195-255 (40) | 0.785 | 0.855 | 0.043 | 0.083 |
|  |  | R: AACGAGCGACACGATAGAGG |  |  |  |  |  |
| Stn382**^2^** | IV | F: CTTGTCCCGGATCATACGC | 151 and 218 bp | - | - | - | - |
|  |  | R: CCCTTAGAGAATTTCCTAGCAG |  |  |  |  |  |
| Gac1125^3^ | XXV | F: **PET-**CATCACACCCAGCCTCTC | 145-200 (24) | 0.835 | 0.872 | 0.02 | 0.042 |
|  |  | R: CCTCCCTCCAACTCTTATCA |  |  |  |  |  |
| Gac2111^3^ | I | F: **FAM-**GTAGAGCACTTGAACTTGAACTG | 209-265 (30) | 0.817 | 0.852 | 0.018 | 0.042 |
|  |  | R: GACGTAGATTGTGGATGTAGAGG |  |  |  |  |  |
| Idh^4^ | XIX | F: TTATCGTTAGCCAGGAGATGG | 271 and 302 bp | - | - | - | - |
|  |  | R: GGGACGAGCAAGATTTATTG |  |  |  |  |  |

**Supporting information 2:** Table with descriptive statistics for all individuals sampled at each sampling site and for the genetically assigned populations (see materials and methods). Abbreviations: n, number of individuals; A number of alleles; H_E_, expected heterozygosity; H_O_, observed heterozygosity. P-values for deviations from Hardy-Weinberg Equilibrium were derived via permutation tests and Bonferroni corrected: ***P<0.001; **P<0.01; *P<0.05.

| Localization | | Chignik | Chignik | Black | Black | Migrants | Hybrids | Fresh- |
| --- | --- | --- | --- | --- | --- | --- | --- | --- |
|  |  | Lagoon | Lake | River | Lake | (1st gen) | (admixed) | water |
|  |  |  |  |  |  |  |  |  |
|  | n | **104** | **122** | **85** | **78** | **34** | **17** | **234** |
| **Locus** |  |  |  |  |  |  |  |  |
| Stn14^1^ | A | 28 | 23 | 27 | 24 | 23 | 15 | 27 |
|  | H_E_ | 0.929 | 0.9** | 0.915 | 0.884 | 0.944 | 0.923 | 0.901*** |
|  | H_O_ | 0.892 | 0.868 | 0.89 | 0.808 | 0.912 | 0.875 | 0.848 |
|  | n | 102 | 121 | 82 | 78 | 34 | 16 | 230 |
|  |  |  |  |  |  |  |  |  |
| Stn61 | A | 26 | 25 | 20 | 19 | 20 | 13 | 24 |
|  | H_E_ | 0.882 | 0.887 | 0.88** | 0.882 | 0.868 | 0.930 | 0.880 |
|  | H_O_ | 0.827 | 0.884 | 0.753 | 0.833 | 0.853 | 0.765 | 0.833 |
|  | n | 104 | 121 | 85 | 78 | 34 | 17 | 233 |
|  |  |  |  |  |  |  |  |  |
| Stn67 | A | 40 | 29 | 23 | 25 | 22 | 16 | 32 |
|  | H_E_ | 0.949 | 0.92 | 0.747 | 0.934 | 0.944 | 0.935 | 0.901 |
|  | H_O_ | 0.952 | 0.865 | 0.858 | 0.924 | 0.935 | 0.875 | 0.837 |
|  | n | 104 | 104 | 75 | 76 | 31 | 16 | 208 |
|  |  |  |  |  |  |  |  |  |
| Stn152 | A | 33 | 23 | 23 | 25 | 22 | 15 | 23 |
|  | H_E_ | 0.93 | 0.925 | 0.931 | 0.935 | 0.942 | 0.920 | 0.921 |
|  | H_O_ | 0.941 | 0.916 | 0.97 | 0.98 | 0.938 | 0.882 | 0.958 |
|  | n | 102 | 119 | 66 | 75 | 32 | 17 | 213 |
|  |  |  |  |  |  |  |  |  |
| Stn175 | A | 21 | 20 | 16 | 18 | 16 | 12 | 19 |
|  | H_E_ | 0.881 | 0.831 | 0.814 | 0.834 | 0.898 | 0.868 | 0.802 |
|  | H_O_ | 0.893 | 0.85 | 0.768 | 0.893 | 0.939 | 0.882 | 0.819 |
|  | n | 103 | 120 | 82 | 75 | 33 | 17 | 227 |
|  |  |  |  |  |  |  |  |  |
| Stn185 | A | 23 | 20 | 21 | 20 | 19 | 15 | 22 |
|  | H_E_ | 0.91 | 0.865 | 0.887 | 0.865 | 0.884 | 0.925 | 0.868 |
|  | H_O_ | 0.833 | 0.832 | 0.869 | 0.831 | 0.848 | 0.938 | 0.836 |
|  | n | 102 | 119 | 84 | 77 | 33 | 16 | 231 |
|  |  |  |  |  |  |  |  |  |
| Stn186 | A | 27 | 22 | 23 | 17 | 15 | 13 | 23 |
|  | H_E_ | 0.9 | 0.796 | 0.794 | 0.816 | 0.903 | 0.909 | 0.763 |
|  | H_O_ | 0.804 | 0.752 | 0.706 | 0.782 | 0.794 | 0.941 | 0.725 |
|  | n | 102 | 121 | 85 | 78 | 34 | 17 | 233 |
|  |  |  |  |  |  |  |  |  |
| Stn211 | A | 13 | 13 | 13 | 12 | 12 | 11 | 13 |
|  | H_E_ | 0.857** | 0.741 | 0.784* | 0.817* | 0.875 | 0.906 | 0.740 |
|  | H_O_ | 0.689 | 0.745 | 0.646 | 0.763 | 0.719 | 0.824 | 0.714 |
|  | n | 103 | 118 | 79 | 76 | 32 | 17 | 224 |
|  |  |  |  |  |  |  |  |  |
| Stn232 | A | 29 | 22 | 24 | 25 | 19 | 15 | 27 |
|  | H_E_ | 0.900 | 0.831 | 0.873 | 0.857 | 0.894 | 0.924 | 0.849 |
|  | H_O_ | 0.900 | 0.852 | 0.821 | 0.896 | 1 | 0.867 | 0.822 |
|  | n | 100 | 118 | 84 | 77 | 33 | 15 | 230 |
|  |  |  |  |  |  |  |  |  |
| Stn265 | A | 36 | 31 | 29 | 31 | 26 | 21 | 36 |
|  | H_E_ | 0.957 | 0.910 | 0.931 | 0.936 | 0.960 | 0.964 | 0.927 |
|  | H_O_ | 0.920 | 0.933 | 0.904 | 0.897 | 0.941 | 1 | 0.904 |
|  | n | 100 | 119 | 83 | 78 | 34 | 17 | 229 |
|  |  |  |  |  |  |  |  |  |
| Stn304 | A | 9 | 7 | 7 | 9 | 7 | 7 | 7 |
|  | H_E_ | 0.665 | 0.631 | 0.716 | 0.708 | 0.582 | 0.712 | 0.672 |
|  | H_O_ | 0.679 | 0.695 | 0.606 | 0.658 | 0.645 | 0.813 | 0.631 |
|  | n | 103 | 117 | 71 | 76 | 31 | 16 | 217 |
|  |  |  |  |  |  |  |  |  |
| Stn319 | A | 31 | 24 | 19 | 17 | 14 | 14 | 31 |
|  | H_E_ | 0.894 | 0.893** | 0.826 | 0.806 | 0.844 | 0.925*** | 0.858*** |
|  | H_O_ | 0.904 | 0.731 | 0.760 | 0.743 | 0.794 | 0.688 | 0.739 |
|  | n | 104 | 119 | 75 | 74 | 34 | 16 | 218 |
|  |  |  |  |  |  |  |  |  |
| Stn1125 | A | 23 | 21 | 18 | 18 | 18 | 15 | 21 |
|  | H_E_ | 0.940 | 0.858 | 0.826 | 0.868 | 0.922* | 0.922 | 0.839 |
|  | H_O_ | 0.931 | 0.818 | 0.760 | 0.831 | 0.848 | 0.824 | 0.799 |
|  | n | 102 | 121 | 75 | 77 | 33 | 17 | 224 |
|  |  |  |  |  |  |  |  |  |
| Stn2111 | A | 24 | 19 | 20 | 15 | 18 | 12 | 17 |
|  | H_E_ | 0.915 | 0.801 | 0.844 | 0.853 | 0.912 | 0.891 | 0.807 |
|  | H_O_ | 0.847 | 0.815 | 0.720 | 0.885 | 0.912 | 0.813 | 0.794 |
|  | n | 98 | 119 | 82 | 78 | 34 | 16 | 228 |
